# Impacts of light limitation on corals and crustose coralline algae

**DOI:** 10.1038/s41598-017-11783-z

**Published:** 2017-09-14

**Authors:** Pia Bessell-Browne, Andrew P. Negri, Rebecca Fisher, Peta L. Clode, Ross Jones

**Affiliations:** 1 Australian Institute of Marine Science, Townsville, QLD, and Perth, WA Australia; 2 The Oceans Institute and The Centre for Microscopy, Characterisation and Analysis, The University of Western Australia, Perth, WA Australia; 3 Western Australian Marine Science Institution (WAMSI), Perth, WA Australia

## Abstract

Turbidity associated with elevated suspended sediment concentrations can significantly reduce underwater light availability. Understanding the consequences for sensitive organisms such as corals and crustose coralline algae (CCA), requires an understanding of tolerance levels and the time course of effects. Adult colonies of *Acropora millepora* and *Pocillopora acuta*, juvenile *P*. *acuta*, and the CCA *Porolithon onkodes* were exposed to six light treatments of ~0, 0.02, 0.1, 0.4, 1.1 and 4.3 mol photons m^−2^ d^−1^, and their physiological responses were monitored over 30 d. Exposure to very low light (<0.1 mol photons m^-2^ d^-1^) caused tissue discoloration (bleaching) in the corals, and discolouration (and partial mortality) of the CCA, yielding 30 d EI_10_ thresholds (irradiance which results in a 10% change in colour) of 1.2–1.9 mol photons m^−2^ d^−1^. Recent monitoring studies during dredging campaigns on a shallow tropical reef, have shown that underwater light levels very close (~500 m away) from a working dredge routinely fall below this value over 30 d periods, but rarely during the pre-dredging baseline phase. Light reduction alone, therefore, constitutes a clear risk to coral reefs from dredging, although at such close proximity other cause-effect pathways, such as sediment deposition and smothering, are likely to also co-occur.

## Introduction

A key to the ecological and evolutionary success of scleractinian corals is the formation of a mutualistic symbiosis with endosymbiotic dinoflagellate microalgae (*Symbiodinium* spp.)^1, 2^. Carbohydrates produced by oxygenic photosynthesis of the algal symbionts and translocated to the coral host provide much of the energy required for maintenance, growth and reproduction^3–5^. This exchange has enabled the symbiosis to survive and coral reefs to proliferate in oligotrophic environments, however, the light dependency has also placed constraints on phototropic corals, limiting their distribution to comparatively low latitudes (~32° north and south of the equator), and shallow depths (~10% of surface light or 50 m)^6–9^.

Benthic light availability is largely determined by surface irradiance (insolation), and primarily influenced by cloud cover, water depth, and transmittance through the water, i.e. water cloudiness or turbidity^10^. Turbidity is mainly affected by suspended sediments^11^, and light attenuation is affected by sediment concentration as well as sediment particle size, shape and colour^12^. Sediments can enter the water column from terrestrial runoff, river plumes, flood water^13–15^, and by natural re-suspension events from currents and wind-driven waves^16, 17^. On a more local scale, sediments can also be released into the water column by dredging and dredging related activities (such as spoil disposal)^18^.

The effects of turbidity on benthic light availability have been quantified for natural resuspension events^10^, flood plumes^19, 20^, and most recently dredging projects^21^. For dredging, shallow (5–10 m depth) habitats close to dredging activities were found to routinely experience complete darkness, sometimes for up to several days in a row^21, 22^. However, a more common feature was extended periods of extreme low light levels (i.e. caliginous or twilight periods)^22, 23^. For example, the daily photosynthetically active radiation (PAR) close to a large dredging operation averaged <10 µmol photons m^−2^ s^−1^ over a 30 d period, which equated to a daily light integral (DLI) of ~1 mol photons m^−2^ d^−1^ ^22, 23^. This is far below the light requirements for many shallow-water, tropical coral species^6^, thus light reduction, in addition to other possible effects of suspended sediments and sediment deposition, constitutes a hazard to shallow benthic communities such as corals^21, 24–26^.

Some corals can nevertheless thrive in highly turbid regions where irradiance is frequently attenuated by elevated suspended sediment concentrations (SSCs); however, these corals have adapted over extended (ecological) time frames to low-light conditions and are generally limited to shallow depths (<4 m)^16, 27–30^. Coral communities living in turbid, nearshore areas may also comprise different species compared to offshore, clear-water reefs, and their tolerance may be due to both community composition and physiological adaptations^31, 32^. On shorter time scales of days to weeks, corals can tolerate episodic periods of low light (<1 mol photons m^−2^ d^−1^) through a range of behavioural and physiological responses. These include photoadaptation of the symbionts and changes in the sub-saturation point for photosynthesis^33^, and in some species switching from phototrophic to heterotrophic feeding^34, 35^. Corals can also temporarily rely on energy reserves^36^, rapidly replenishing reserves when conditions become more favourable^37^.

Only a few studies have examined the effects of exposure to very low light (<0.1 mol photons m^−2^ d^−1^) on corals, and these have mostly been associated with investigating the role of the symbiotic dinoflagellates in the symbiosis. For example, Yonge and Nicholls^38^ showed that extrusion of *Symbiodinium*, and subsequent discolouration (bleaching), occurred in response to darkness for a variety of tropical reef flat corals over 18 d (*Lobactis scutaria*), 22 d (*Psammocora contigua*) and 19 d (*Galaxea fascicularis*). Franzisket^39^ exposed four species of hermatypic corals (*Pocillopora elegans*, *Porites compressa*, *Montipora verrucosa* and *Fungia scularia*) to darkness for 60 d. All colonies bleached within 10–20 d and there was no growth observed over the exposure period^39^. *Pocillopora elegans* died after 30 d while the remaining species survived over the exposure period^39^. Kevin and Hudson^40^ showed the temperate coral, *Plesiastrea urvillei*, lost algal symbionts after ~40 d in darkness. Hoegh-Guldberg and Smith^41^ observed bleaching of *Stylophora pistillata* in the dark after 10 d, while Titlyanov, *et al*.^42^ observed bleaching of *S*. *pistillata* after 4 d. In a study investigating the mechanism of bleaching, DeSalvo, *et al*.^43^ reported colonies of *Acropora palmata* and *Montastraea faveolata* becoming pale and eventually bleaching after 3–5 d in darkness.

A temporary reduction in benthic light is a well-known hazard of dredging-related activities^24^. We recently demonstrated that light attenuation represents a greater threat to coral health than any physical effects of suspended sediment particles^44^. The study investigated the impacts of three light levels (~0, 1.1 and 8.3 mol photons m^−2^ d^−1^), and three suspended sediment concentrations (0, 30 and 100 mg L^−1^), on three common coral species, including *Acropora millepora*, *Porites* spp. and *Montipora capricornis*; and found bleaching of corals in low light treatments (~0 and 1.1 mol photons m^−2^ d^−1^) and no mortality associated with 100 mg L^−1^ of suspended sediments when light levels remained high (8.3 mol photons m^−2^ d^−1^). This result demonstrated the importance of light reduction on coral health and the need to identify low light thresholds to improve the management of future sediment generating activities undertaken in the vicinity of coral reefs^44^. The risk to nearby coral reef communities could be better predicted and managed if there was a clearer understanding of the associated physiological effects, along with the time-frame of any effects, and tolerance limits of key benthic organisms.

To that end, we investigated here the thresholds for light reduction on adult colonies of *A*. *millepora* and *Pocillopora acuta*, juvenile (7 month old) colonies of *P*. *acuta*, and on the crustose coralline alga (CCA) *Porolithon onkodes*. Like corals, CCA are essential structural components of coral reef ecosystems^45–47^, and provide chemical cues for settlement of many benthic invertebrate larvae, including corals^48–50^. However, their response to extended periods of low light (<1 mol photons m^−2^ d^−1^) has not been investigated. The aim of the study is to understand the response thresholds, and time-course of the response, of high light adapted adult and juvenile corals, and CCA, to extended periods of low light relevant to conditions generated by offshore dredging^22, 23^. To contextualise the results of the laboratory based study, results are discussed with respect to temporal and spatial changes in benthic light availability recently described for a large scale capital dredging project^22, 23^.

## Results

Fragments of 2 coral species (*A*. *millepora*, and *P*. *acuta*), along with juvenile *P*. *acuta* (7 months old) and a species of crustose coralline alga (*P*. *onkodes*) were exposed to 6 light treatments (~0, 0.02, 0.1, 0.4, 1.1 and 4.3 mol photons m^−2^ d^−1^). Non-destructive techniques were used to monitor coral health throughout the exposure period, including image analysis of coral colour and photochemical efficiency (*F*_v_/*F*_m_) of algal symbionts. At the end of the exposure period photosynthetic incubations were conducted to determine the photosynthetic capacity of the corals. Irradiance-response relationships were then determined to identify thresholds of low light conditions to guide the management of future dredging and other sediment generating activities.

### Health parameters assessed through time

**Figure 1:**
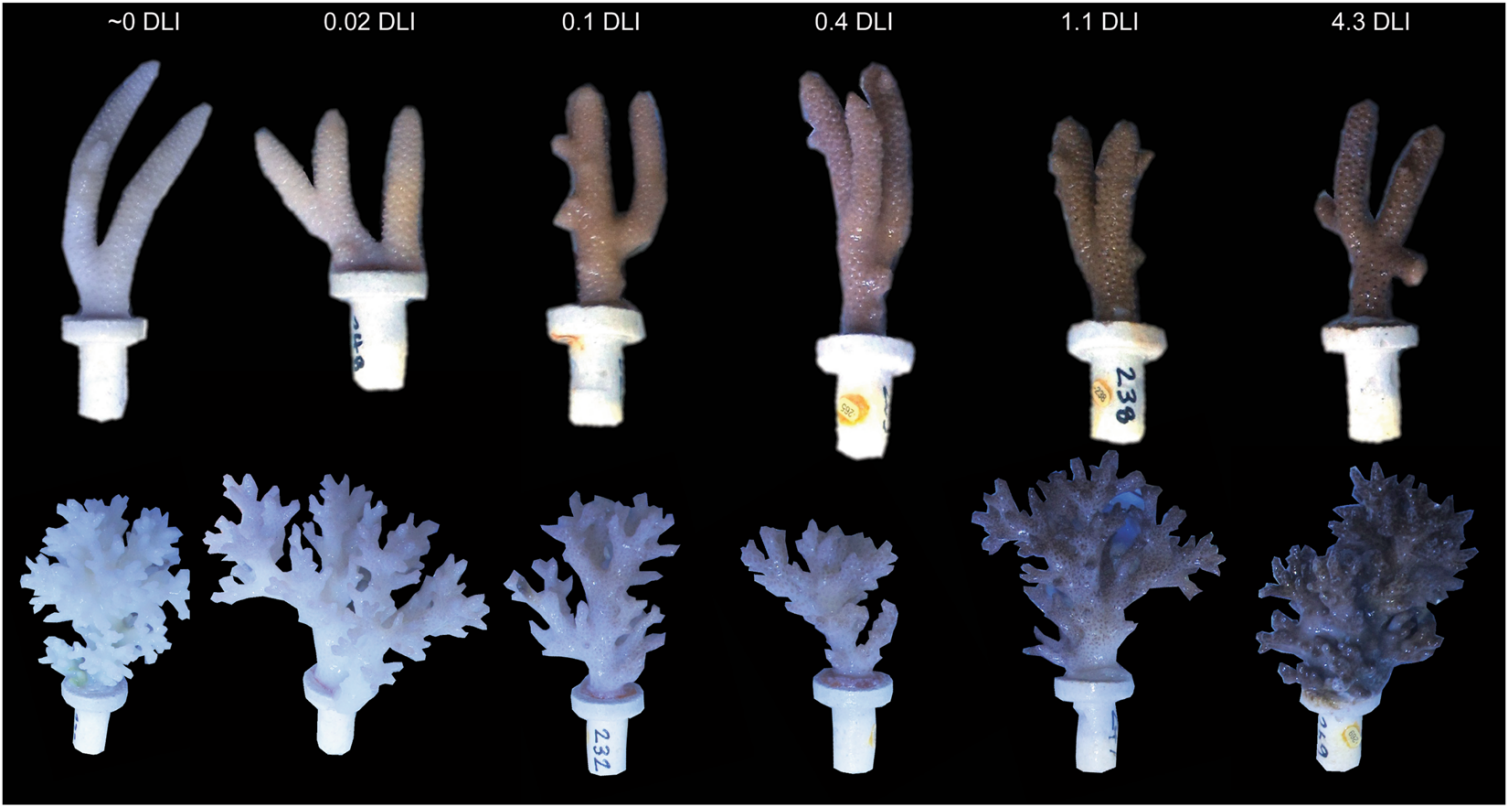
Photographs of representative *A*. *millepora* and *P*. *acuta* fragments after 30 d of exposure to the six daily light integral (DLI) irradiance treatments of ~0, 0.02, 0.1, 0.4, 1.1 and 4.3 mol photons m^−2^ d^−1^.

Coral colonies in the lower light treatments gradually lost colour though time, with paling observed after 10 d in all groups when exposed to <0.1 mol photons m^−2^ d^−1^. By 20 d corals exposed to <0.1 mol photons m^−2^ d^−1^ were bone white, while those exposed to 0.4 mol photons m^−2^ d^−1^ were very pale. This colour loss was uniform across each fragment. At the end of the exposure period there was a clear gradation in colour from fully pigmented in the 4.3 mol photons m^−2^ d^−1^ treatment, to bone white in the ~ 0 mol photons m^−2^ d^−1^ treatment (Fig. 1). CCA were dark red at the start of the exposure period and through time this colour intensified in fragments exposed to 0.4 and 1.1 mol photons m^−2^ d^−1^. Those CCA fragments exposed to ≤0.1 mol photons m^−2^ d^−1^ discoloured rapidly and had sections of pale tissue and sections of bone white skeleton where cells had been lost.

Light treatment (DLI), time of exposure (Time) and species (Species) all strongly influenced the measured coral health parameters (partial mortally, colour index (a proxy for bleaching), and maximum quantum yield (*F*_*v*_/*F*_*m*_)) (Table 1). There was strong evidence (AICc weights all near 1, Table 1) that a full three way interaction between these predictors was the best model to describe the changes observed.

**Table 1:** Top generalised linear mixed effect model fits for each health parameter measured through time (partial mortality, colour index and *F*_v_/*F*_m_), including number of parameters (*n*), Akaike information criterion (AICc), δ AICc, model weights and R^2^ values. See Supplementary Information Tables S1–S3 for the complete full subsets output.

Parameter	Model	*n*	AICc	δ AICc	AICc weight	R^2^
Partial mortality	DLI × Time × Species	51	1965.7	0	1	0.34
Colour index	DLI × Time × Species	51	1973.8	0	0.97	0.56
*F*_*v*_/*F*_*m*_	DLI × Time × Species	51	2087.7	0	1	0.72

**Figure 2:**
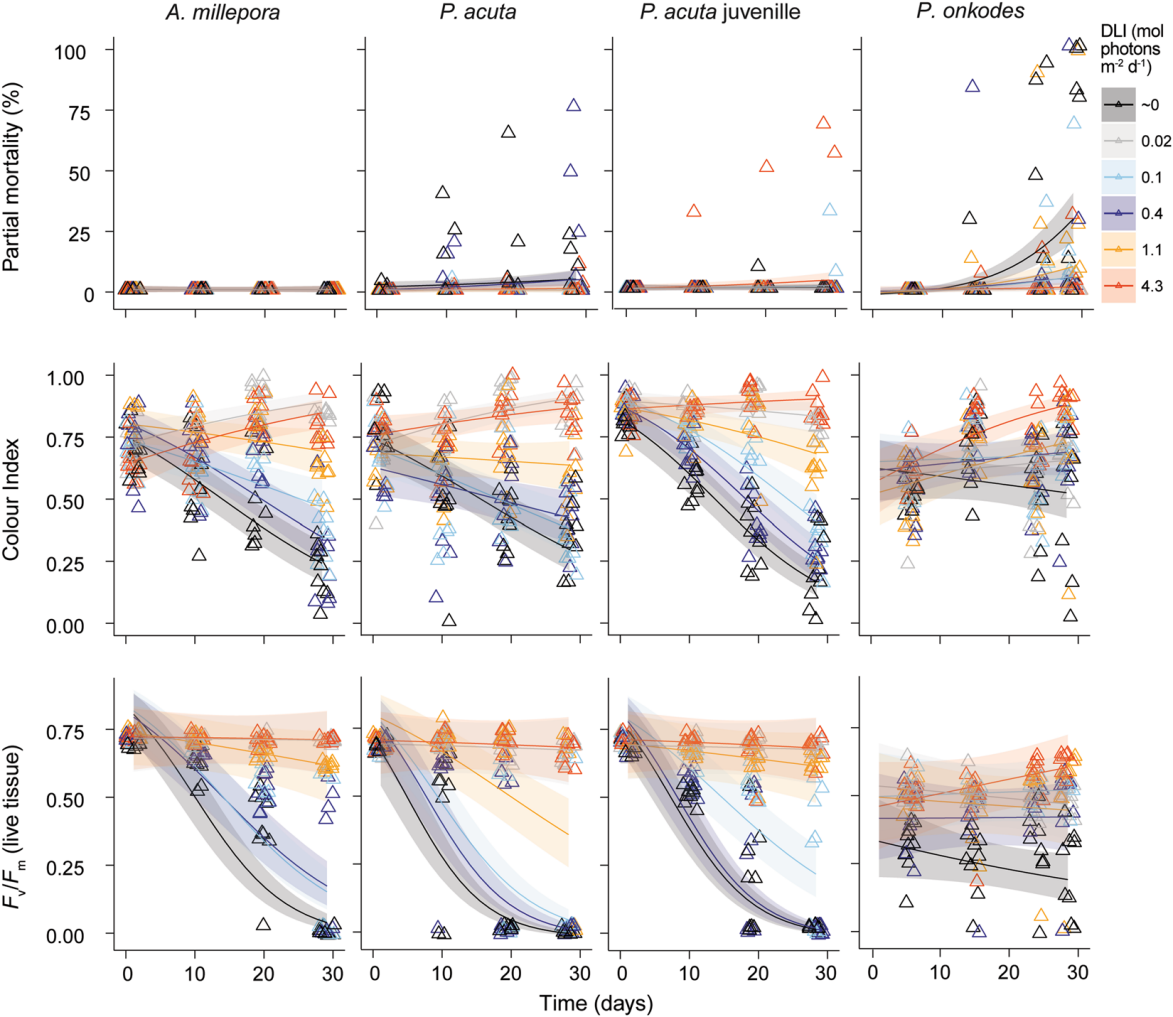
Partial mortality, colour index and maximum quantum yield (*F*_v_/*F*_m_) of the corals *A*. *millepora*, *P*. *acuta* adults, *P*. *acuta* juveniles and the crustose coralline alga *P*. *onkodes*, for the 6 light treatments of ~0, 0.02, 0.1, 0.4, 1.1 and 4.3 DLI (mol photons m^−2^ d^−1^), as indicated by the colours. Raw data are presented (triangles), along with curves showing best-model fitted relationships (lines) and corresponding 95% credible intervals (ribbons).

Partial mortality was observed in juvenile and adult *P*. *acuta* as well as in *P*. *onkodes* during the light limitation exposures, while *A*. *millepora* showed no signs of tissue loss regardless of light intensity, even after 30 d of exposure (Fig. 2). *P*. *acuta* adults suffered from more partial mortality than juveniles, and tissue loss was apparent in the ~0 and 0.1 mol photons m^−2^ d^−1^ treatments after 10 d (Fig. 2). The mortality observed in juvenile *P*. *acuta* was inconsistent across treatments, with highest morality occurring for the second highest treatment (1.1 mol photons m^−2^ d^−1^), suggesting this mortality may not be associated with low light conditions of the experimental treatments (Fig. 2). *P*. *onkodes* exhibited the highest level of partial mortality, with the majority apparent after 25 d in the ~0–0.4 mol photons m^−2^ d^−1^ treatments, while no mortality was observed at 4.3 mol photons m^−2^ d^−1^ (Fig. 2).

The tissue colour index of corals declined in the light treatments ≤0.4 mol photons m^−2^ d^−1^ and this response became more pronounced over time, especially in the darkness treatment (~0 mol photons m^−2^ d^−1^) (Fig. 2). There was also a subtle increase in pigmentation in the two highest light treatments (≥1.1 mol photons m^−2^ d^−1^) (Fig. 2). The colour index of *P*. *onkodes* varied throughout the experiment, with pigmentation lowest after 30 d in the dark treatment (~0 DLI) (Fig. 2).

A gradual decline in *F*_v_/*F*_m_ was observed for *A*. *millepora* colonies in each of the light treatments from the first 10 d, with the most pronounced decreases observed in the light treatments ≤0.1 mol photons m^−2^ d^−1^ (Fig. 2). *F*_v_/*F*_m_ also declined in adult *P*. *acuta* colonies exposed to 0.4 mol photons m^−2^ d^−1^. The *F*_v_/*F*_m_ of those corals in the darkest exposures (≤0.02 mol photons m^−2^ d^−1^) were 0, as few *Symbiodinium* spp. remained in these treatments after 20 d. The *F*_v_/*F*_m_ of *P*. *acuta* juveniles also declined over the 30 d in the ≤0.1 mol photons m^−2^ d^−1^ treatments (Fig. 2). *P*. *onkodes* had reduced *F*_v_/*F*_m_ after five d of exposure to treatment conditions, while *F*_v_/*F*_m_ were lowest in the ~0 DLI treatment and remained stable in all other treatments (Fig. 2).

### Photosynthetic Incubations

**Figure 3:**
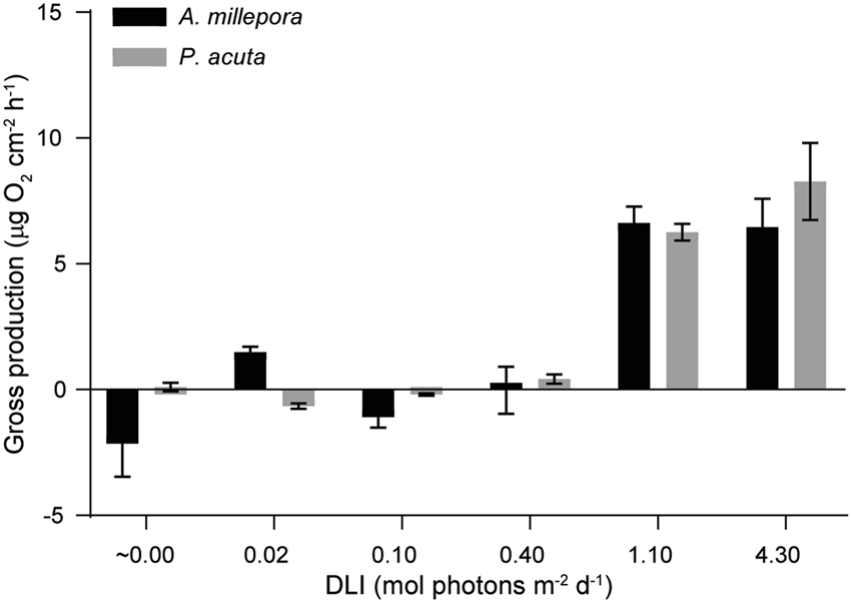
Gross photosynthesis (negative values indicate respiration) at saturating irradiance (419 µmol photons m^−2^ s^−1^), determined from photosynthesis irradiance (P–I) curves for both *A*. *millepora* (black) and adult *P*. *acuta* (grey) colonies across the 6 light treatments (~0, 0.02, 0.1, 0.4, 1.1 and 4.3 mol photons m^−2^ d^−1^). Data presented are mean ± SE, *n* = 3.

Photosynthetic incubations at the end of the exposure period at the saturating irradiance of 419 µE m^−2^ s^−1^ (determined from photosynthesis irradiance curves), revealed limited to no photosynthetic capacity in adult coral colonies exposed to a DLI of ≤0.4 mol photons m^−2^ d^−1^ (Fig. 3, Supplementary Fig. S1). Fragments that were exposed to <1.1 mol photons m^−2^ d^−1^ for the duration of the experiment displayed no increase in oxygen production or photosynthetic capacity (Fig. 3).

### Irradiance-response relationships

**Figure 4:**
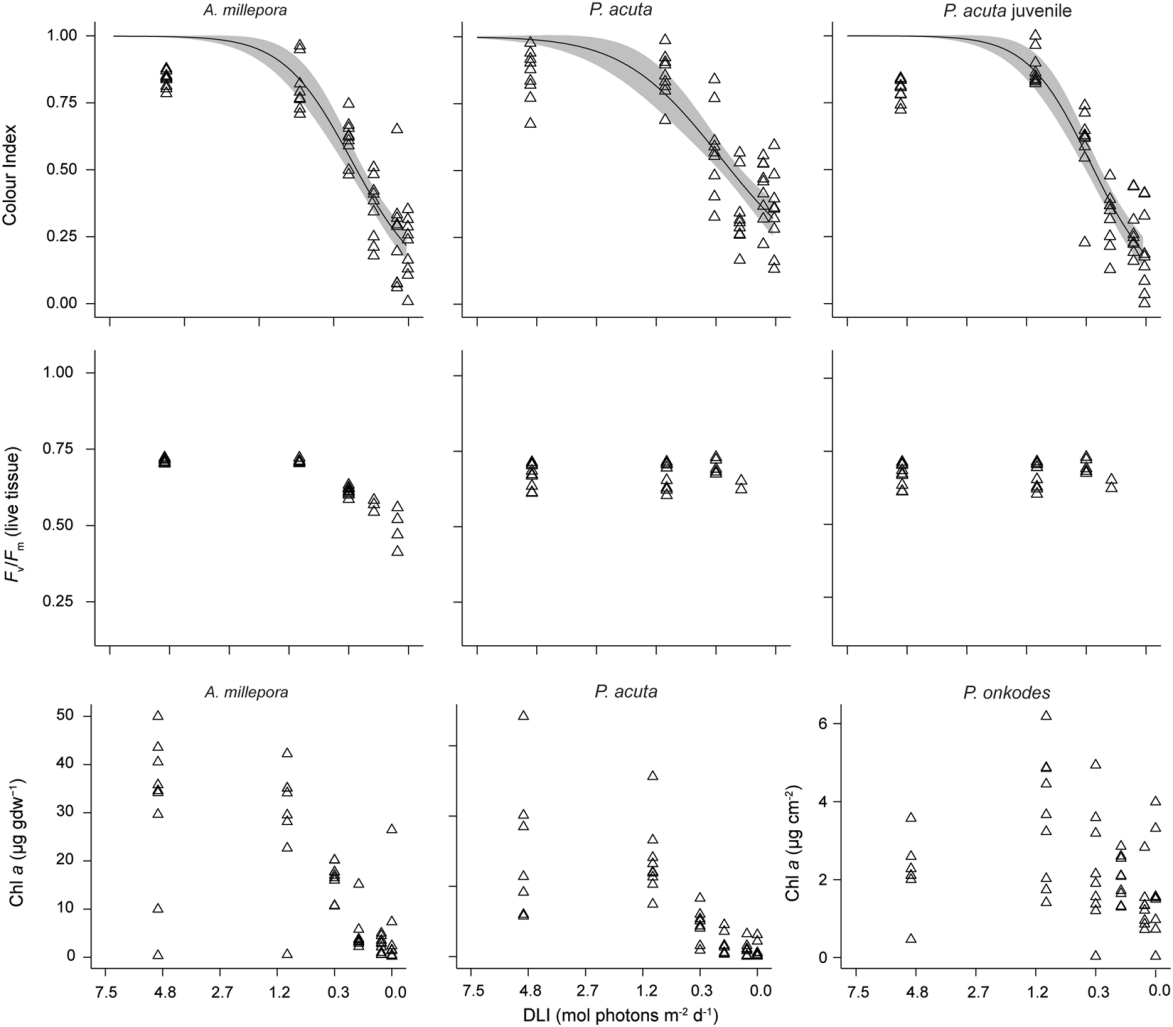
Irradiance-response relationships for colour index and maximum quantum yield (*F*_v_/*F*_m_), and Chl *a* concentrations of corals *A*. *millepora*, *P*. *acuta* adults, *P*. *acuta* juveniles and the crustose coralline alga *P*. *onkodes*, after 30 d of exposure to 6 light treatments of 0, 0.02, 0.1, 0.4, 1.1 and 4.3 mol photons m^−2^ d^−1^ (note inverse DLI values on x-axis). Raw data are presented (triangles), with modelled relationships (lines) and 95% confidence intervals (ribbons). See Supplementary Information Figs S2 and S3 for relationships after 10 and 20 d.

Patterns of mortality were not observed in *A*. *millepora* or *P*. *acuta* juveniles, however, increased mortality with decreased light exposure after 30 d were apparent in *P*. *acuta* and *P*. *onkodes* (Supplementary Fig. S2). Patterns of decreasing colour index with reduced light exposure were clear, and were similar across all species, with *A*. *millepora* and *P*. *acuta* juveniles exhibiting the lowest colour index values (Fig. 4). Similar trends were observed for *F*_v_/*F*_m_ in *A*. *millepora* (Fig. 4). The patterns observed after 20 d, particularly for *F*_v_/*F*_m_ were more well defined than after 30 d, and relationships after 10 d showed less clear patterns again (Supplementary Fig. S3). Trends in Chl *a* content increased with increasing light exposures, reaching a maximum around 1.1 mol photons m^−2^ d^−1^ (Fig. 4), and correlations were observed between colour index and Chl *a* for all coral species at 30 d (Supplementary Fig. S4). No clear trends in any of the sub-lethal health parameters were detected for the CCA *P*. *onkodes*.

The effect threshold for irradiance EI_10_ was defined as irradiance which elicited a 10% effect on mortality, colour, *F*_v_/*F*_m_ or Chl *a* content. A higher EI_10_ (or EI_50_) indicates a more sensitive response (the effect was reached with less light attenuation). After 10 d exposure, the sub-lethal impacts associated with even the lowest light treatment represented less than a 10% change, meaning that an EI_10_ threshold could not be calculated (i.e. even complete loss of light did not cause a 10% decline in colour index or *F*_v_/*F*_m_). After 20 d, the threshold irradiances for colour index in the corals (also a proxy for 10% bleaching) ranged from EI_10_ 0.39–1.4 mol photons m^−2^ d^−1^ (Table 2). After 30 d, the EI_10_s for colour index increased to 1.2–1.9 mol photons m^−2^ d^−1^ indicating the bleaching thresholds had been reached with less attenuation. The EI_50_ values (a proxy for 50% coral bleaching) occurred under conditions of greater light attenuation and ranged from 0.16–0.23 mol photons m^−2^ d^−1^ after 30 d (Table 2).

**Table 2:** Nonlinear regression (four-parameter logistic function) results of proportional mortality, colour index and maximum quantum yield (*F*_v_/*F*_m_) for *A*. *millepora*, *P*. *acuta* adults and *P*. *acuta* juveniles after 10, 20 and 30 d of exposure to treatment conditions. Both the 10% and 50% effect irradiances (EI_10_ and EI_50_) are presented as mol photons m^−2^ d^−1^ with 95% confidence intervals. A higher EI_10_ (or EI_50_) indicates a more sensitive response (the effect was reached with less light attenuation). No EI_10_ and EI_50_ effects were observed at day 10 for any species, and the irradiance thresholds for Chl *a* reductions could not be determined using non-linear regressions. NE indicates no effect for the given level of change (10% or 50%).

Health variable	Species	Day 20		Day 30	
		EI_10_	EI_50_	EI_10_	EI_50_
Proportional mortality	*A*. *millepora*	NE	NE	NE	NE
	*P*. *acuta*	NE	NE	1.50 (0.69,2.63)	NE
	*P*. *acuta* juv	NE	NE	NE	NE
	*P*. *onkodes*	NE	NE	8.99 (2.56,19.33)	1.44 (0.23,3.65)
Colour Index	*A*. *millepora*	0.86 (0.35,1.6)	0.04 (0.00,0.17)	1.4 (1.0, 1.9)	0.23 (0.17,0.30)
	*P*. *acuta*	1.4 (0.69,2.3)	NE	1.9 (1.1, 2.8)	0.16 (0.09,0.24)
	*P*. *acuta* juv	0.39 (0.25,0.56)	0.02 (0.01,0.04)	1.2 (0.83, 1.6)	0.22 (0.16,0.28)
*F*_v_/*F*_m_	*A*. *millepora*	NE	NE	1.4 (0.77,2.30)	0.36 (0.22,0.53)
	*P*. *acuta*	1.2 (0.44,2.30)	0.26 (0.13,0.44)	NE	NE
	*P*. *acuta* juv	NE	NE	NE	NE

### Thresholds of low light relative to dredge related water quality conditions

**Figure 5:**
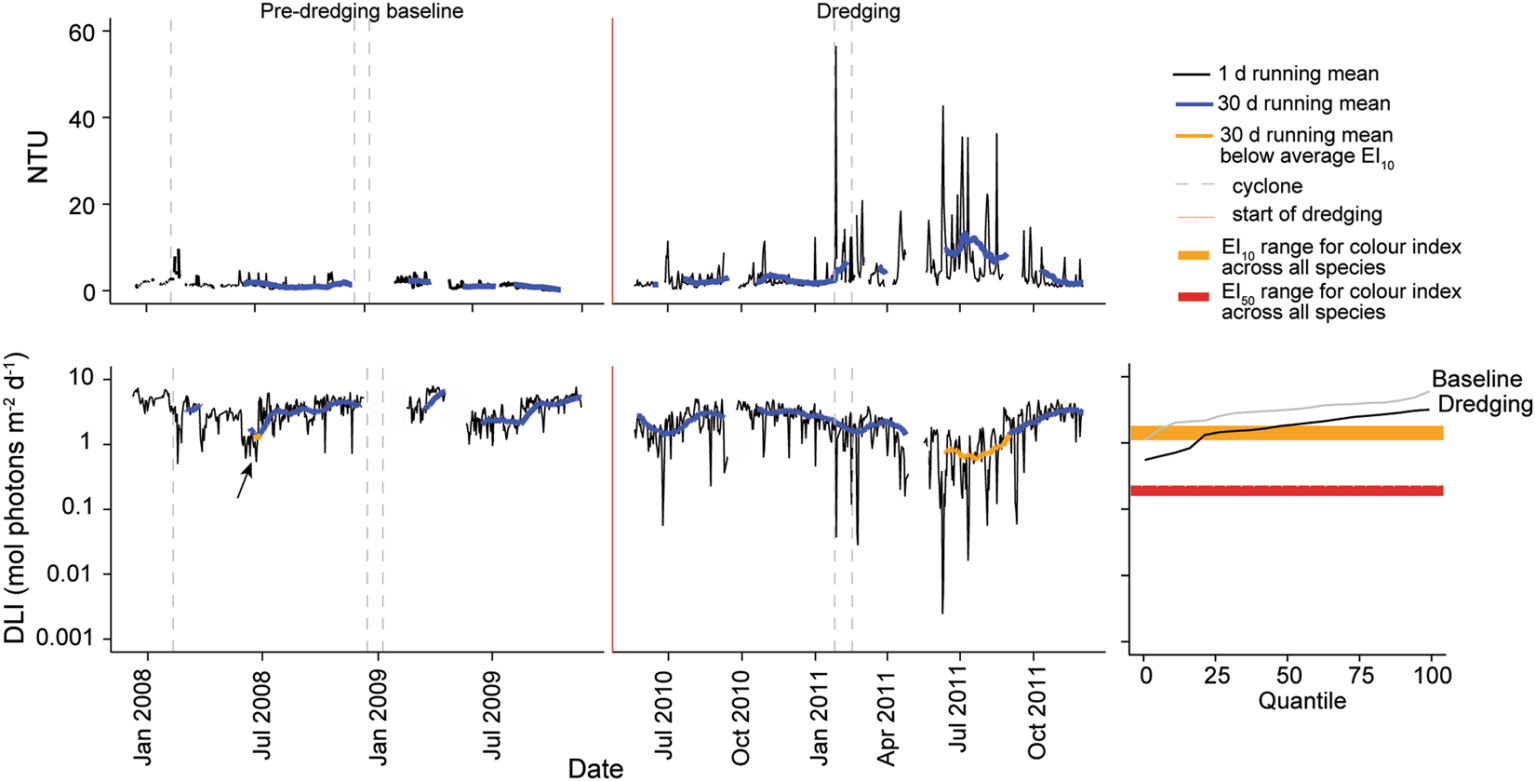
Water quality conditions before and during a large-scale, capital dredging project on the coral reefs surrounding Barrow Island (north-west Australia) where ~7.6 M m^3^ of sediment was removed over a 530 d period (for further details see Jones, *et al*.^23^ and Fisher, *et al*.^22^). Turbidity (NTU) and benthic PAR levels (DLI, mol photons m^−2^ d^−1^) are shown for the extended pre-dredging baseline and dredging phase at one site, located 0.8 km from the primary excavation site at 6.2 m depth. Horizontal coloured ribbons on the right hand quantile plot show the range of calculated DLI thresholds for each of the 3 species and life stages (see Table 2) investigated, including *A*. *millepora*, *P*. *acuta* and *P*. *acuta* juveniles for both 10 and 50% impacts on colour index (EI_10_ = orange and EI_50_ = red). Data presented are the daily mean NTU and DLI (black), with 30 d running means (blue solid line). Periods when the 30 d running mean DLI drops below the experimentally calculated threshold values (Table 2) are indicated by the appropriate threshold colour. Quantiles of 30 d running mean DLI are also presented across the pre-dredge (grey) and dredging (black) phases.

During the 336 d pre-dredging baseline phase at Barrow Island, mean daily turbidity levels at a site 0.8 km from the excavation activities was typically very low (0–10 NTU), and the DLI ranged between 1 and 8 mol photons m^−2^ d^−1^ (Fig. 5). The 30 d running mean DLI only dropped below the average EI_10_ bleaching threshold for all species (Table 2) for a short period in June of 2008 (see arrow Fig. 5). During the 530 d dredging phase turbidity was much higher (0–50 NTU), and daily irradiance correspondingly lower, associated with plumes of resuspended sediment moving over the monitoring site (Fig. 5). Days with very low light levels (i.e. <0.1 mol photons m^−2^ d^−1^) were more common, and the 30 d running mean DLI was very low towards the end of the dredging period, when elevated NTUs combined with low winter insolation levels (Fig. 5). Cyclones during the baseline and dredging phases had noticeable short term effect on light availability, but did not result in light reduction below the EI_10_ threshold value on a 30 d running mean scale (Fig. 5).

Quantiles of the 30 d running mean periods indicate that during the pre-dredging phase, the EI_10_ threshold is exceeded approximately 10% of the time for *P*. *acuta* adults (~34 d) and approximately 2% of the time in *A*. *millepora* and *P*. *acuta* juveniles (~8 d) (Fig. 5). During the dredging, these quantile values are higher, with *P*. *acuta* adults exceeding the EI_10_ approximately 50% of the time (~265 d), while for *A*. *millepora* and *P*. *acuta* juveniles this threshold is exceeded approximately 25% of the time (~132 d) (Fig. 5). Light levels over 30 d were not low enough to reach the EI_50_ values for these species (Fig. 5, Table 2).

## Discussion

Exposure to extended periods of low irradiance and darkness had considerable impacts on the health of high light adapted coral and CCA. Corals (both adult and juvenile) became bleached, losing colour and Chl *a* content. At the end of the exposure period corals in the very low light treatments (<1 mol photons m^−2^ d^−1^) were heavily bleached (bone-white) signifying loss of algal symbionts. Tissue loss (partial mortality) was observed in adult *P*. *acuta* after prolonged periods of near darkness (<0.4 mol photons m^−2^ d^−1^). The CCA *P*. *onkodes*, was also sensitive to light-limitation showing discoloration and partial mortality. The 30 d EI_10_ thresholds for bleaching in the corals (mean irradiance which results in a 10% change in colour) was 1.2–1.9 mol photons m^−2^ d^−1^. Underwater light levels measured during a dredging project on a shallow (~6 m), tropical, clear water reef (see Fisher, *et al*.^22^ and Jones, *et al*.^23^), were found to routinely fall below this value in close proximity (~800 m away) from a working dredge. Light levels rarely fell below the threshold during the pre-dredging baseline phase. This study demonstrates that light reduction alone constitutes a clear risk to clear-water coral reefs from dredging, although at such close proximity other cause-effect pathways, such as sediment deposition and smothering, are likely to also occur.

The gradual loss of colour and eventual bleaching of corals exposed to low light (<1 mol photons m^−2^ d^−1^) are consistent with studies which exposed corals to complete darkness. In this study corals began noticeably paling after 4–5 days and were heavily bleached after 10 days, similar to the observations of impacts caused by complete darkness reported by Hoegh-Guldberg and Smith^41^, Franzisket^39^, Yonge and Nicholls^38^ and Titlyanov, *et al*.^42^, for a range of reef flat species, but slower than observed by DeSalvo, *et al*.^43^, who observed heavy bleaching of *Acropora palmata* and *Montastraea faveolata* after 3 and 5 d in darkness respectively. While these studies provide important thresholds determining the time required to bleach in complete darkness, our study provides critical light thresholds for bleaching that can be applied to manage dredging that causes near-darkness for weeks^22, 23^.

Low light thresholds for adult and juvenile corals varied. Juvenile *P*. *acuta* were slightly more resilient than the adults, however, as the difference was small (30 d EI_10_ for colour loss of 1.2 versus 1.9 mol photons m^−2^ d^−1^ respectively), the thresholds developed for adult corals would be applicable to 7 month old recruits. *P*. *acuta* colonies were far more sensitive to low light (<1 mol photons m^−2^ d^−1^) conditions in terms of partial colony mortality than *A*. *millepora*, where no mortality occurred. The relative sensitivity contradicts what might be expected when considering the life history traits of these two species, with *A*. *millepora* considered a competitive species, while *P*. *acuta* is an early colonising, weedy species and, therefore, anticipated to be more resilient to stressors^51^.

Several microsensor studies have shown that when placed in darkness, coral tissue rapidly (within minutes) enters a hypoxic and then near anaerobic state^52–55^. This is due to high metabolic activity of the symbiotic dinoflagellates and polyp tissue, limiting the diffusive supply of O_2_ from the surrounding water through the diffusion boundary layer. Although corals routinely enter hypoxia at night time, tissue oxygen concentrations also rapidly increase on exposure to light in the early morning^53^. How corals tolerate hypoxia is unknown, although symbiotic anemones have been found to survive through fermentation processes involving glycolysis^56–58^. Such fermentation processes have been observed in corals when exposed to hypoxia from sediment smothering^59^. These processes produce ATP at approximately 6-fold lower yields than aerobic respiration^60^, offering a short term, temporary energy source, but not over extended periods in low light (<1 mol photons m^−2^ d^−1^).

A characteristic of the patterns of low-light induced bleaching was the uniform, even, tissue discolouration (Fig. 1), as opposed to the often variegated and sunlight orientated patterns of discolouration that can occur during warm-water bleaching events^61^. This suggests a different mechanism of bleaching, but the cue that initiates the dissociation is not clear. In *A*. *millepora*, and *P*. *actua* adults and juveniles, the quantum efficiency *F*_v_/*F*_m_ of the *Symbiodinium* spp. decreased following long periods in darkness and the very low-light treatments (<0.4 mol photons m^−2^ d^−1^). This could be due to unstacking and structural changes of the thylakoid membrane, leading to reduced electron transport^43, 62^. A reduction in the translocation of photosynthate from the algal symbionts to the host has been suggested as a potential cue for warm bleaching^63, 64^. Alternatively, if the hypoxia of the coral tissues in very low light is related to the metabolic activity of the symbionts in the coral tissues, then elimination of the source of the problem, the algal symbionts (i.e. bleaching), seems a relatively simple explanation and survival strategy. Irrespective of the underlying mechanism, towards the end of the exposure period the loss of algal symbionts at daily light integrals lower than <1.1 mol photons m^−2^ d^−1^ resulted in photosynthesis:respiration ratios of less than one, demonstrating little photosynthetic capacity. As the colonies were not fed during the exposure period, they were most likely drawing on energy reserves to meet their metabolic requirements^3, 65^.

It is possible to contextualise the results from these laboratory-based studies using information from a recent detailed analyses of spatial and temporal patterns of benthic light availability measured before and during a large scale, capital dredging project on a clear-water reef (at Barrow Island, ~50 km offshore of the Pilbara coast of north-west Western Australia^22, 23^). Extended periods of low light naturally occurred during the pre-dredging period in the (austral) winter time, associated with shorter days, lower solar declination and probably increased cloud cover, but the E_10_ thresholds for discolouration were rarely reached. However, during the dredging phase, there were marked reductions in benthic light availability, and a site 800 m from the dredging routinely experienced periods of daytime darkness, darkness over the whole day (defined as <0.04 mol photons m^−2^ d^−1^), and darkness for 1 to 6 consecutive days^22^. These periods, again, occurred during the winter time when turbid plumes combined with seasonal light minima. Although the hazard associated with dredging turbidity and light reduction have been known since the 1970s^24^, these results better describe the risk and the spatial context associated with the elevated turbidity. Under prolonged periods of light reduction, some corals could survive by switching from phototrophic to heterotrophic feeding to maintain a positive energy balance^3, 34, 35, 65^, or temporarily draw on energy reserves^36^, replenishing them when light conditions become more favourable^37^.

CCA was more sensitive to the impacts of low irradiance than both adult and juvenile corals, and suffered from higher levels of partial mortality. However, patterns of colour loss and decreases in maximum quantum yields (*F*_*v*_/*F*_*m*_) with light limitation were not as clearly defined as those in corals, with reductions apparent almost immediately followed by stability over time. While some CCA species are well adapted to low light levels in the mesophotic zone^66^, the results presented here suggest that high light adapted species, such as *P*. *onkodes*, are not able to readily adapt to periodic low light exposure. Loss of CCA has implications for reef health, as it is one of the most important and widespread reef-builders in the marine photic zone worldwide and provides important cues for the settlement of coral larvae^45, 48–50^. Changes in CCA abundance can therefore influence the structure and function of coral reef ecosystems^45^. Reduced prevalence of CCA associated with low light conditions during dredging may result in declines in coral recruitment, potentially exacerbating issues associated with increased deposited sediments interfering with coral settlement^67^. Clearly more work is needed to understand the potential changes associated with dredging for key non-coral biota, such as CCA, and the persistence and recovery time associated with CCA decline resulting from exposure to low light conditions.

The thresholds for effects of light limitation on corals and CCA identified here are likely to be conservative as they did not take into account potential shifts in available spectra that may occur under dredging plumes. Light attenuation through a plume of suspended sediments will be characterised by a shift in spectra towards yellow and green wavelengths, that are less useful for photosynthesis^21^. It is possible that the impacts on coral and CCA health by light attenuation caused by sediment plumes would be more severe than reported in the current study, where shifts in spectra to less useful wavelengths were not applied. Furthermore, light attenuation is only one of several potential stressor pathways related to sediment plumes caused by dredging^21, 25, 26^. Although suspended particles are not likely to significantly exacerbate the impacts of shading in turbid waters^44^, elevated SSCs needed to significantly attenuate light in shallow water (<10 m) tropical reef environments will most likely also result in appreciable levels of sediment deposition, which in turn can smother corals resulting in tissue necrosis^21, 59, 68, 69^. In addition, periods of high light attenuation from dredging are likely to be dispersed with periods of less attenuation^23^, which may alleviate some of the negative impacts of long exposures to poor water quality and/or long dredging campaigns. The compounding effects of decreased light quality and quantity, elevated SSCs, and deposited sediment as well as the periodic nature of these stressors clearly require further investigation.

In summary, these results provide light limitation thresholds for high light adapted, shallow water corals and CCA; as well as insights into both direct and indirect pathways associated with the effects of dredging on the physiology of corals and the ecology of coral reefs. Exposure to a DLI of ~1.5 mol photons m^−2^ d^−1^ for a period of 30 d caused a 10% decline in the health of corals and partial mortality in some species (i.e. *P*. *acuta*), and exposure to less than ~0.2 mol photons m^−2^ d^−1^ resulted in a 50% decline. The principle physiological response was dissociation of the coral-algal symbiosis, a well-known sub-lethal stress response of corals^70^. Indirect ecological effects include reducing the health of CCA which provide important cues for the settlement of coral larvae^49^. Water quality programs designed to reduce impacts on reefs during dredging campaigns should recognise the potential for the effects of elevated turbidity to combine with annual light minima to reduce light levels below identified required minima for corals and CCA. The effects of other dredging related water quality pressures, such as sediment deposition also need to be considered. The light attenuation scenarios presented here (high light adapted corals and CCA exposed to low light conditions) represents one of several scenarios, and more work is needed to clarify the sensitivity of a wider range of reef-building corals and CCA to low light periods experienced during dredging and natural re-suspension events so turbidity-generating activities, such as dredging, can be managed to effectively protect these ecologically important taxa.

## Methods

Experiments were conducted using adults of two hard coral species *Acropora millepora* (Ehrenberg 1834) and *Pocillopora acuta* (Lamarck 1816), juvenile (7 month old) *P*. *acuta* colonies and the crustose coralline algae (CCA) *Porolithon onkodes* (Penrose & Woelkerling 1992). Eight colonies and subsequent genotypes of *A millepora*, *P acuta* and *P*. *onkodes* that were free of biofouling and disease were collected from 3–10 m in lagoonal area of Davies Reef and Broadhurst Reef (both mid-shelf reefs centrally located in the Great Barrier Reef (GBRMPA permits G12/35236.1 and G13/35758.1) and transferred to the Australian Institute of Marine Science (AIMS) National Sea Simulator (SeaSim) at Cape Cleveland near Townsville Queensland. Corals and CCA were fragmented into replicates with a surface area of ~10 cm^2^ *A*. *millepora* and *P*. *acuta* and ~3 cm^2^ *P*. *onkodes*, and fragments were glued onto aragonite coral plugs. The fragments were then held in 200 L flow-through holding tanks in the SeaSim for 6 weeks to recover from the collection and preparation procedures. During the holding period, corals and CCA were exposed to a 12-h light:dark (L:D) cycle comprising of a 2 h period of gradually increasing light in the morning (06:00–8:00 h), 8 h of constant illumination at 200 µmol photons m^−2^ s^−1^, and a 2 h period of gradually decreasing light in the afternoon (16:00–18:00 h). Our previous study indicated no effect of irradiance on the bleaching of *A*. *millepora* between 1 and 8 mol photons m^−2^ d^−1^ daily light integral (DLI, or total summed PAR) over 28 h^44^ and corals and CCA in the present system experienced an intermediate DLI of 7.2 mol photons m^−2^ d^−1^, consistent with the typical range at the site of collection (4 – 8 mol photons m^−2^ d^−1^).

*P*. *acuta* juveniles were reared from parent colonies collected at Davies Reef. Colonies that were free of biofouling and diseases were kept in the SeaSim and larvae were collected monthly over the summer. These larvae were left to settle on small aragonite plugs using chips of CCA to induce settlement^49^. Recruits were then left to develop over 7 months before being transferred to the same holding tanks as adult fragments for the 6 week healing and light acclimatisation period.

All experiments were conducted over a 30 d period in clear PVC tanks holding 49 L of filtered seawater. In this experiment we did not feed the corals in order to mimic the impacts of an offshore dredging scenario where heterotrophic feeding is less important, and where the carbonate sediments from dredging contain very low levels of organic carbon^21^ and are not a useful source of energy. Our previous study exposing corals to both low light and carbonate sediments demonstrated that only low light affected coral health^44^. In tank circulation was maintained with a TUNZ pump (EcoTech Marine, PA, US). Seawater was fed into each tank at 800 mL min^−1^ (resulting in ~6 complete water turnovers d^−1^). Water temperature was maintained at 26 ± 0.5 °C, and salinity at 33 ± 0.5‰ throughout the experiment. Above each tank two Sol White LED lights (Aquaria Illumination, IA, US) were suspended to ensure even illumination throughout the tank.

For the dark treatments, the tanks were covered in black corrugated plastic sheets (to reduce light contamination) and for the remaining five light treatments the corals were exposed to a 12-h L:D cycle composed of a 6 h period of gradually increasing light in the morning (06:00–12:00 h), and a 6 h period of gradually decreasing light in the afternoon (12:00–18:00 h). Light levels were measured at the depth of the corals using an underwater spherical quantum sensor (Li-COR LI-193). Over the course of the day the corals experienced DLIs of 0, 0.02, 0.1, 0.4, 1.1 or 4.3 mol photons m^−2^. For the 5 light treatments, their maximum intensity was 1, 5, 20, 50 and 200 µmol photons m^−2^ s^−1^ respectively. Corals held in darkness did experience very low level light exposure (albeit for a few minutes) during weekly photographing (see below), and thus the treatment is hereafter referred to as a DLI of ~0 mol photons m^−2^. Three tank replicates were used for each treatment and three replicates of each species per tank were used for general health assessments, with different genotypes randomly allocated amongst tanks. Light levels throughout the exposure period were measured as 0.00 ± 0.00, 3.68 ± 0.83, 7.25 ± 0.67, 12.11 ± 0.66, 45.44 ± 0.60 and 167.50 ± 1.35 µmol photons m^−2^ s^−1^ (all mean ± standard error) in the 0, 0.02, 0.1, 0.4, 1.1 and 4.3 mol photons m^−2^ d^−1^ treatments respectively (Supplementary Fig. S4). These light regimes were selected based on analyses of benthic light levels measured during a large-scale, capital dredging project on the coral reefs surrounding Barrow Island (north-west Australia) where ~7.6 Mm^3^ of sediment was removed over a 530 d period (for further details see Jones, *et al*.^23^ and Fisher, *et al*.^22^).

All species were photographed every 10 d using a high resolution digital camera and the camera settings and the surrounding light environment kept the same during the photographing process over the duration of the experiment. Changes in colour were assessed weekly from the photographs. Images were analysed with the image processing software program ImageJ^71^, using the histogram function on a selection of representative live tissue, taking the arithmetic mean of pixel values (range 0–255) on a black and white scale. At the end of the experiment, these were standardised to the maximum and minimum values for each species, and converted to a range between 0 and 1. During the photographing process, any partial mortality of the corals was noted and quantified from the photographs using ImageJ. We previously demonstrate a good correlation between colour index and Chl *a* concentration^44^.

Chlorophyll fluorescence of the endosymbiotic dinoflagellate algae within tissue of each coral fragment was measured using a mini-PAM fluorometer (Walz, Germany). Measurements were obtained using a 6 mm fibre-optic probe positioned perpendicular to the coral fragment and 3 mm away (controlled by a rubber spacer). Initial fluorescence (*F*_0_) was determined by applying a weak pulse-modulated red light (650 nm, ~0.15 μmol photons m^−2^ s^−1^). Maximum fluorescence (*F*_m_) was then measured following a saturating pulse of light. Maximum quantum yield (*F*_v_/*F*_m_) is the proportion of light used for photosynthesis by chlorophyll when all reaction centres are open^72^ and is determined by the following equation:

$$\frac{{F}_{{\rm{v}}}}{{F}_{{\rm{m}}}}=({F}_{{\rm{m}}}-{F}_{0})/{F}_{{\rm{m}}}$$

Coral fragments were dark-adapted for 30 min prior to measuring the yield. Fluorescence data were collected before the experiment began, and after 10, 20 and 30 d. Measurements were only taken over live tissue, and 1–4 measurements were taken and averaged per fragment, depending on live tissue available.

Oxygen respirometry was conducted using a system of 8 sealed, clear, perspex chambers with a magnetic stir bar that were submerged in a jacket of running water to buffer temperature fluctuations (maintained between 25.5 and 26.5 °C). Of the 8 chambers, 6 contained coral fragments, while the other two were seawater blanks (to correct for seawater production/respiration). Each chamber was fitted with an oxygen spot (OXSP5, Pyroscience, Germany) and connected to a fibre-optic oxygen meter (Firesting O_2_, Pyroscience, Germany), which had been calibrated to 100 and 0% O_2_. Above the chambers two Sol White LED lights (Aquaria Illumination, IA, US) were suspended to ensure even illumination. The chambers were exposed to 8 discrete light intensities (0, 10, 30, 75, 150, 300, 480 µmol photons m^−2^ s^−1^) for between 15 and 30 minutes each. Surface area and volume of each coral skeleton were determined using wax dipping and volume displacement for standardisation.

All data were analysed with R software (version 3.2.3, R Core Team^73^). The relative influences of environmental factors on coral health parameters were assessed using a full subsets model selection approach^74^, where models were compared with Akaike Information Criterion with corrections for sample size (AICc) and R^2^. The models with the lowest AICc (within 2) and the fewest parameters was chosen as the ‘best’ model. For modelling of relationships, tank and coral fragment identity were included as random factors. For health parameters assessed through time (partial mortality, *F*_v_/*F*_m_ and colour index) a logit transformation was used with generalised linear mixed models to determine the impacts of health parameters for each species. Each health dataset was explored using the protocol described by Zuur, *et al*.^75^. For modelling of relationships, species, time and DLI were included as fixed factors. Gaussian generalised linear mixed models were fit with the package lme4^76^. Full subsets comparison was completed using dredge in the MuMIn package^77^. The final model was re-fit using MCMC to allow calculation of error terms using the R2jags package^78^. Chl *a* concentrations were modelled with a Tweedie distribution using the cplm package^79^.

Pressure-response relationships were examined after 10, 20 and 30 d across the six light levels for each species and health parameter. These relationships were not modelled for partial mortality as effect irradiance calculations (i.e. EI_10_, EI_50_) would be meaningless without reaching full fragment mortality, instead we modelled the probability of observing any mortality using a binomial distribution. The remaining health parameter (colour index, maximum quantum yield and Chl *a*) relationships were fitted with drc package^80^. Models were fitted as 4-parameter logistic regressions where the data showed a clear trend in responses across light treatments, with 10 and 50% impact levels subsequently determined. 10 and 50% impact levels were calculated in comparison to the controls (4.3 mol photons m^−1^ d^−1^ exposure at day 0).

Hyperbolic tangent functions were fitted to incubation gross production data for both *A*. *millepora* and adult *P*. *acuta* fragments.

## Electronic supplementary material

Supplementary Information
