# Supplementary material for: Impacts of light limitation on corals and crustose coralline algae
Source: Sci Rep. 2017 Sep 14;7:11553. doi: 10.1038/s41598-017-11783-z (PMC5599546; doi:10.1038/s41598-017-11783-z)
Supplement: Supplementary file 1 — Supplementary Information [file 41598_2017_11783_MOESM1_ESM.pdf]

Supplementary information: Impacts of light limitation on corals and crustose coralline algae

Pia Bessell-Browne[1,2,3], Andrew P. Negri[1,3], Rebecca Fisher[,1,3], Peta L. Clode[2], Ross Jones[1,3]

[1]*Australian Institute of Marine Science, Townsville, QLD, and Perth, WA, Australia*

[2] *The Oceans Institute and The Centre for Microscopy, Characterisation and Analysis, The University of Western Australia, Perth, WA, Australia*

[3] *Western Australian Marine Science Institution (WAMSI), Perth, WA, Australia*

* corresponding author email: piabessellbrowne@gmail.com

Table S1. All model fits for mortality measured through time, including the number of parameters (*n*), Akaike information criterion (AICc), δ AICc, model weights and R^2 values.

Model	*n*	AICc	R^2	AIC weight
Species + DLI	20	-2665.02	0.063	1
Time × Species + DLI	28	-2649.84	0.146	0
Species + DLI + Time	22	-2629.93	0.063	0
Time × Species	18	-2375.93	0.165	0
Species	10	-2371.79	0.065	0
Species + Time	12	-2328.7	0.065	0
DLI × Time × Species	98	-1239.92	0.32	0
DLI × Species	50	-1192.34	0.153	0
DLI × Species + Time	52	-1185.62	0.153	0
DLI + Time × Species	28	1585.634	0.132	0
DLI × Time	26	1657.208	0.099	0
DLI + Time	16	1670.122	0.051	0
DLI	14	1686.644	0.030	0
Time	8	2210.801	0.052	0
1	4	2273.53	0.000	0

Table S2. All model fits for colour index measured through time, including the number of parameters (*n*), Akaike information criterion (AICc), δ AICc, model weights and R^2 values.

Model	*n*	AICc	R^2	AIC weight
DLI × Time × Species	98	2090.209	0.601	1
DLI × Time	26	2263.021	0.395	0
DLI + Time × Species	28	2301.576	0.383	0
DLI × Species + Time	52	2331.142	0.364	0
DLI × Species	50	2333.155	0.318	0
Time × Species + DLI	28	2367.063	0.383	0
Time × Species	18	2384.487	0.145	0
DLI + Time	16	2475.077	0.287	0
DLI	14	2519.275	0.239	0
Time	8	2528.007	0.048	0

Species + Time	12	2535.854	0.064	0
1	4	2567.648	0.000	0
Species + DLI	20	2576.497	0.256	0
Species + DLI + Time	22	2614.853	0.303	0
Species	10	2648.175	0.017	0

Table S3. All model fits for maximum quantum yield measured through time, including the number of parameters (*n*), Akaike information criterion (AICc), δ AICc, model weights and R^2 values.

Model	*n*	AICc	R^2	AIC weight
DLI × Time × Species	98	1658.108	0.730	1
DLI × Species + Time	52	2066.207	0.291	0
DLI × Species	50	2110.019	0.272	0
DLI × Time	26	2197.068	0.526	0
Species + DLI + Time	22	2318.069	0.189	0
Time × Species + DLI	28	2348.296	0.190	0
DLI + Time × Species	28	2358.579	0.192	0
Species + DLI	20	2368.408	0.170	0
DLI + Time	16	2404.583	0.226	0
DLI	14	2423.829	0.209	0
Time × Species	18	3199.217	0.267	0
Time	8	3404.609	0.184	0
Species	10	3444.669	0.020	0
Species + Time	12	3568.297	0.205	0
1	4	3572.229	0.000	0

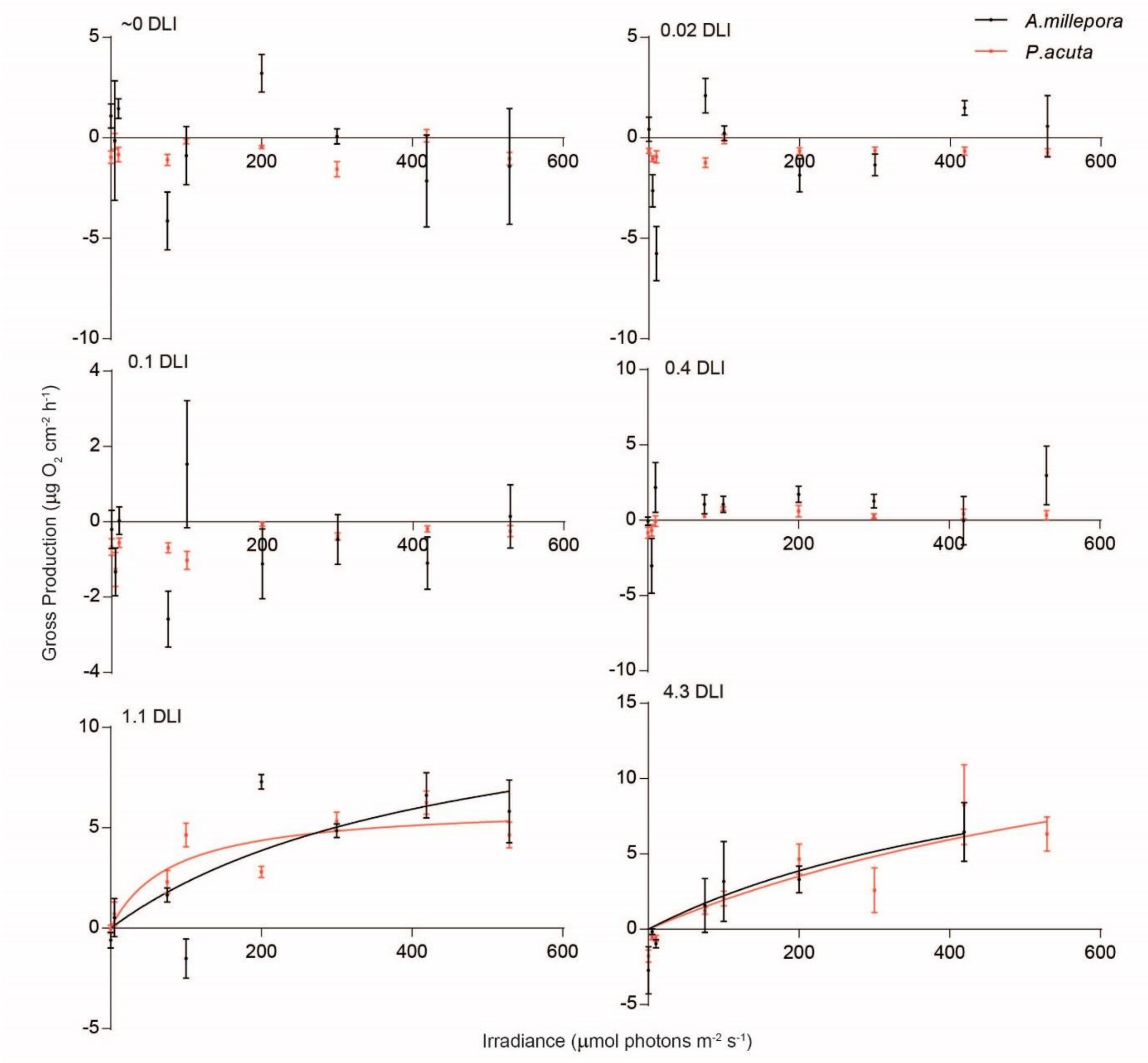

Figure S1. Photosynthesis-irradiance (P-I) curves created using a hyperbolic tangent function for *A. millepora* and adult *P. acuta* fragments across the 6 light treatments (0, 0.02, 0.1, 0.4, 1.1 and 4.3 DLI mol photon m^{-2}).

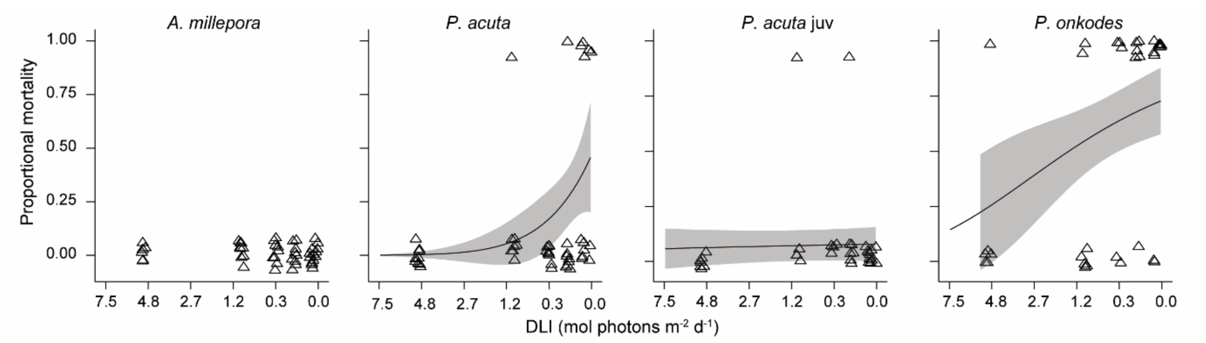

Figure S2. Pressure-response relationships for mortality of *A. millepora*, *P. acuta* adults, *P. acuta* juveniles, after 30 d of exposure to 6 light treatments of 0, 0.02, 0.1, 0.4, 1.1 and 4.3 DLI (mol m^{-2} d^{-1}). Note inversions on both axes.

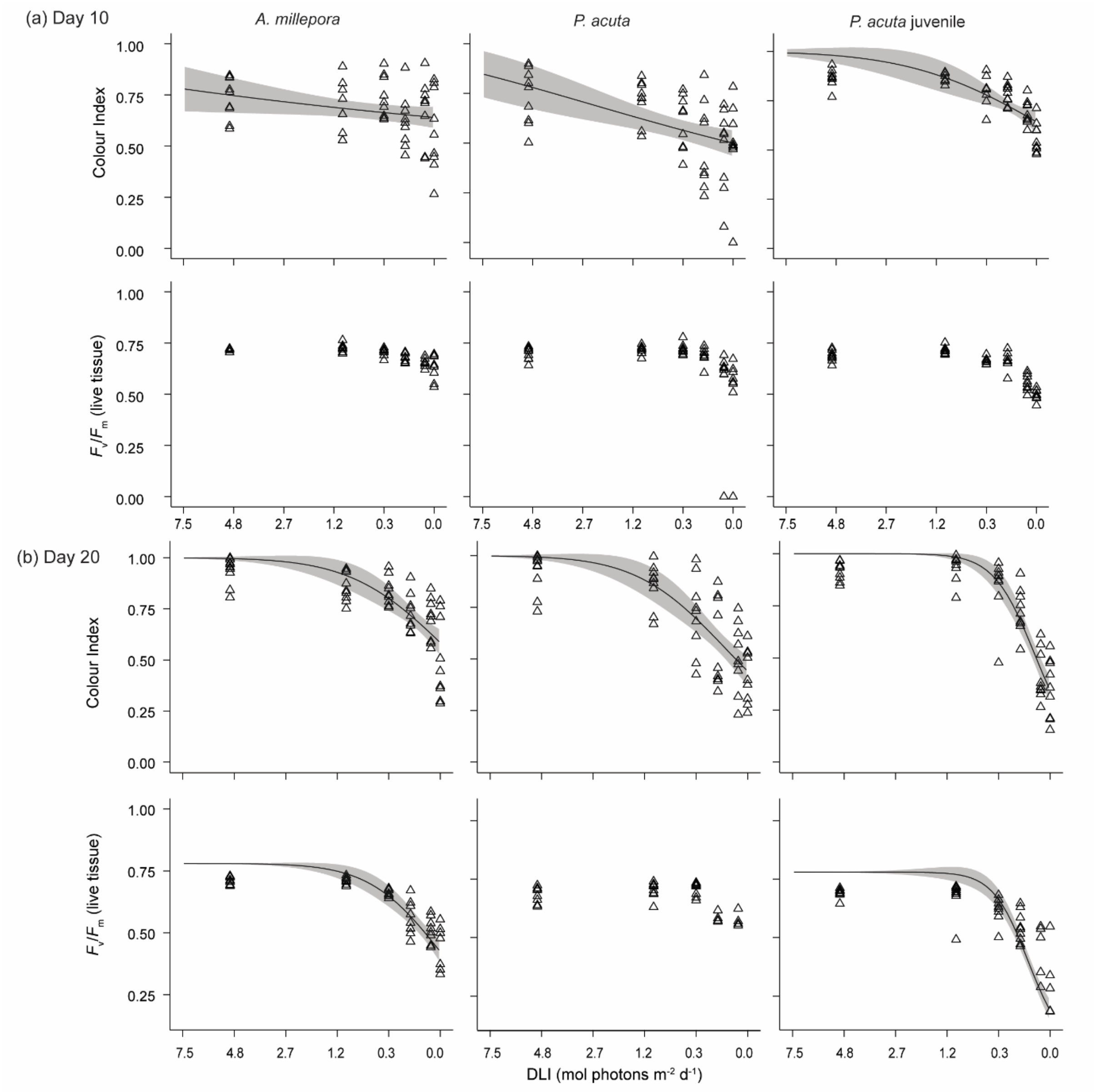

Figure S3. Pressure-response relationships for colour index and maximum quantum yield (F_v/F_m) of *A. millepora*, *P. acuta* adults, *P. acuta* juveniles, after (a) 10 and (b) 20 d of exposure to 6 light treatments of 0, 0.02, 0.1, 0.4, 1.1 and 4.3 DLI (mol m^{-2} d^{-1}).

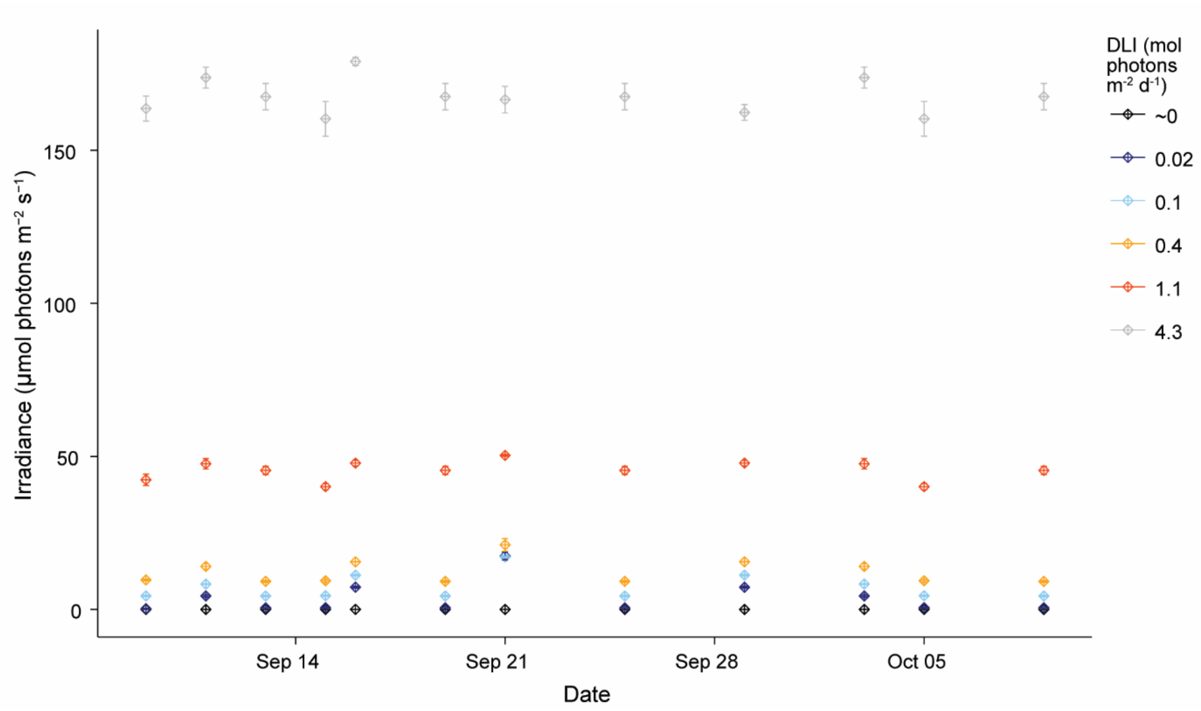

Figure S4. Light levels throughout the duration of the 30 d exposure period averaged across each light treatment. Data presented is mean ± standard error.
